# Supplementary material for: Ag–thiolate interactions to enable an ultrasensitive and stretchable MXene strain sensor with high temporospatial resolution
Source: Nat Commun. 2024 Jun 25;15:5354. doi: 10.1038/s41467-024-49787-9 (PMC11200319; doi:10.1038/s41467-024-49787-9)
Supplement: Supplementary file 1 — Supplementary Information [file 41467_2024_49787_MOESM1_ESM.pdf]

Supporting information for
Ag-Thiolate Interactions to Enable an Ultrasensitive and Stretchable MXene Strain Sensor with High Temporospatial Resolution

Yang Liu[#,*1], Zijun Xu[#,1], Xinyi Ji[#,2], Xin Xu[1], Fei Chen[1], Xiaosen Pan[1], Zhiqiang Fu[1], Yunzhi Chen[1], Zhengjian Zhang[1], Hongbin Liu[1], Bowen Cheng*[,1], Jiajie Liang*[2,3,4]

[1] State Key Laboratory of Biobased Fiber Manufacturing Technology, Tianjin Key Laboratory of Pulp and Paper, Tianjin University of Science and Technology, Tianjin 300457, China

[2] School of Materials Science and Engineering, National Institute for Advanced Materials, Nankai University, Tianjin 300350, China.

[3] Key Laboratory of Functional Polymer Materials of Ministry of Education, College of Chemistry, Nankai University, Tianjin 300350, China.

[4] School of Materials Science and Engineering & Smart Sensing Interdisciplinary Science Center, Nankai University, Tianjin 300350, China.

Address correspondence to: liuyangtust@tust.edu.cn (Y. Liu), bowen15@tiangong.edu.cn (B. Cheng), liang0909@nankai.edu.cn (J. Liang)

[#] These authors contributed equally to this work.

This PDF file includes:

Supplementary Note 1 and 2

Supplementary Fig. 1 to 25

Supplementary Table 1

Supplementary Movie 1

Supplementary References

Supplementary Note 1:

Figure S5 plots the relative change in resistance of the S-M/A_1 strain sensor with various thicknesses (T) of 2.4, 1.4, and 0.7 μm as a function of applied strain. Obviously, the stretchability of the S-M/A_1 strain sensor increases with the device thickness decreases from thickness of 2.4 to 1.4 μm. The highest GF for the rigid S-M/A_1 strain sensor with device thickness of 2.4 μm was calculated to be 445000 in working strain range of 9-11%. Although the maximum GF drops to 71400 as the device thickness declines to 1.4 from 2.4 μm, the working strain range shows a significant increase from 11% to 45%. This stretchability increase can be attributed to the fact that reducing thickness in the film can render the brittle thin film flexible[1]. With the thickness further decreased to 0.7 μm from 1.4 μm, the stretchability of the S-M/A_1 strain sensor decreased and the GF values increased. This phenomenon was mainly attributed to easier destruction of electrical junctions between adjacent sensing islands under stretching when the sensing film had a very small thickness[2].

Supplementary Note 2:

We conducted tensile and crack distribution analysis on the model using a standard module of ABAQUS software. The simulation process included the following four steps: (1) Material definition. We utilized the Johnson-Cook damage model and referenced stress-strain curves from previous reports for MXene[3] and AgNW[4]. The fracture condition was set based on the tensile properties of MXene and AgNW. The density, elastic modulus, and tensile strength used in the simulation process for MXene (and S-MXene) was 4.4 g/cm^3, 80 GPa, and 0.67 GPa[3,5], and 10.5 g/cm^3, 86 GPa, and 2 GPa[4,6], respectively. (2) Contact settings. We then specified frictionless and adhesive contact between AgNW and MXene (or S-MXene). The calculation of Ag-S bonding points was based on the MPTES molecule weight and S-MXene mass, with the adhesion strength defined as 17.74 kcal·mol^{-1}. (3) Mesh partitioning. Tetrahedral free mesh partitioning with C3D10 elements for MXene (or S-MXene) and AgNW models was employed. (4) Boundary conditions. We fixed the constraints at one end and the load tensile strain of 10% at the other end.

Supplementary figures and tables

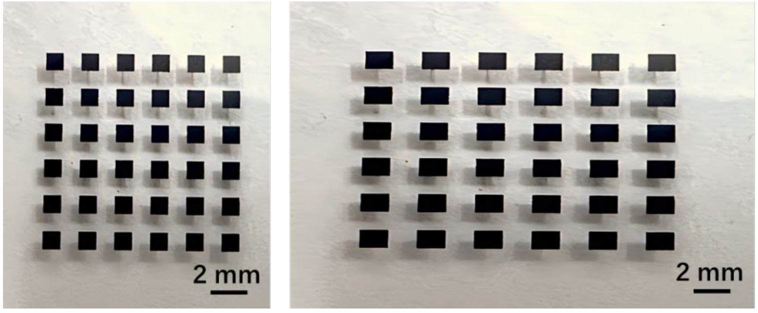

Supplementary Fig. 1. S-M/A_1 sensor array in the unstretched state and under 60% strain.

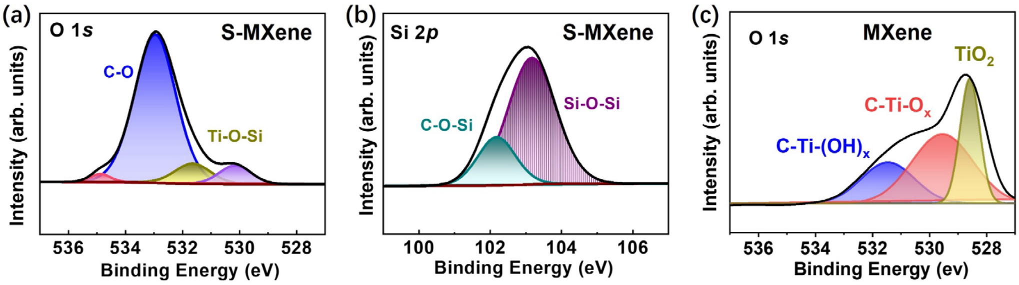

Supplementary Fig. 2. XPS spectra of a, b) S-MXene and c) MXene films in the O 1*s* and Si 2*p* region.

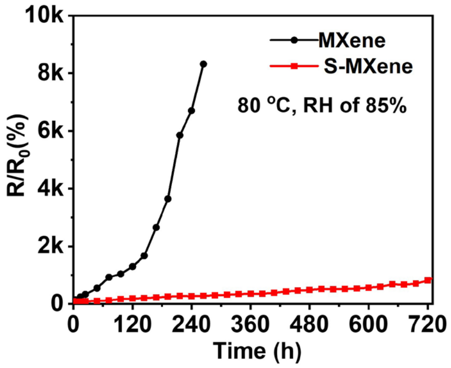

Supplementary Fig. 3. Relative resistance changes of MXene and S-MXene after storage in the aging chamber with a temperature of 80 °C and relative humidity (RH) of 85% for 30 days. Compared with the MXene film, the S-MXene film exhibited much improved oxidation resistance.

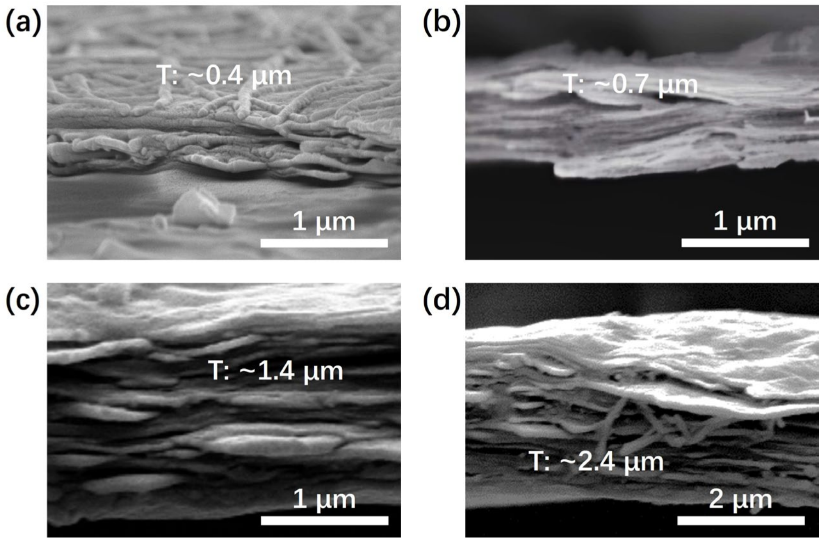

Supplementary Fig. 4. S-M/A sensing films with different thicknesses (T).

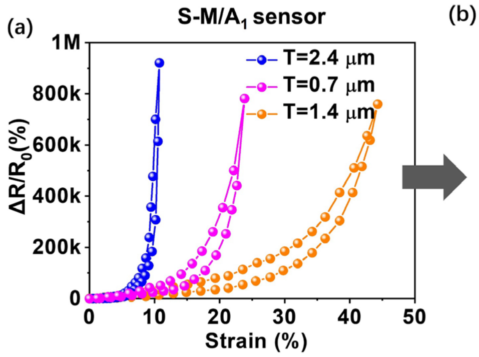

(b)

Thickness(um)	Range(%)	GF
T=1.4	0-21	1970
	21-30	7800
	30-38	22600
	38-45	71400
T=0.7	0-10	2480
	10-18	10200
	18-22	56100
	22-24	226700
T=2.4	0-5	2590
	5-8	25300
	8-9	93200
	9-11	445000

Supplementary Fig. 5. (a) Relative resistance variation as a function of large strain and its details for S-M/A_1 sensors with different thickness. (b) The calculated GF for the S-M/A_1 sensors with different thickness.

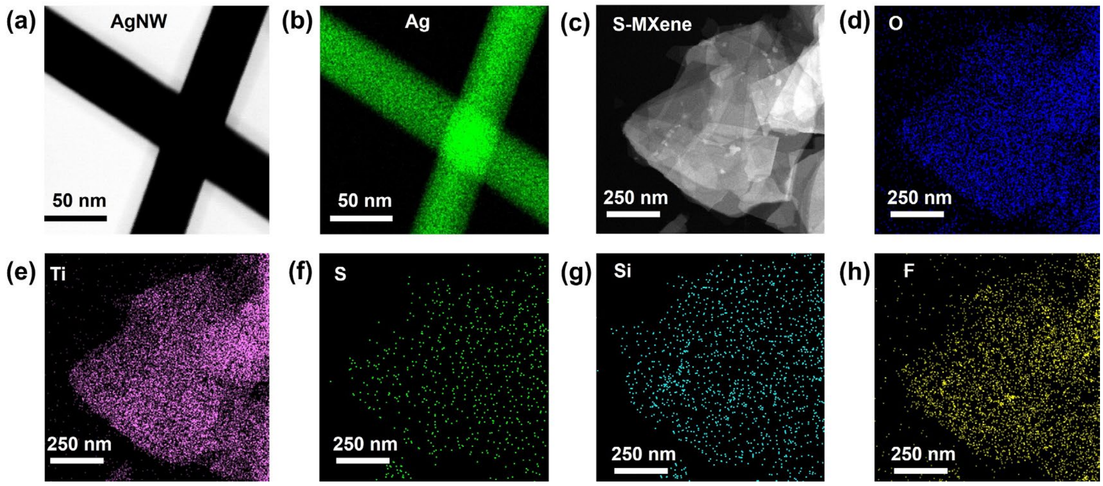

Supplementary Fig. 6. TEM images and corresponding EDS element maps of (a) and (b) AgNW and (c)-(h) S-MXene.

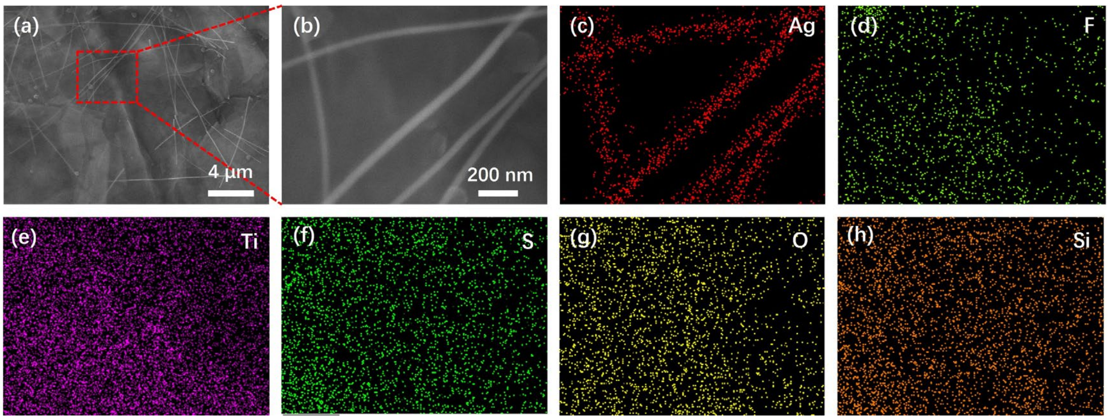

Supplementary Fig. 7. SEM images of original S-M/A film under 0% strain and corresponding elemental distribution.

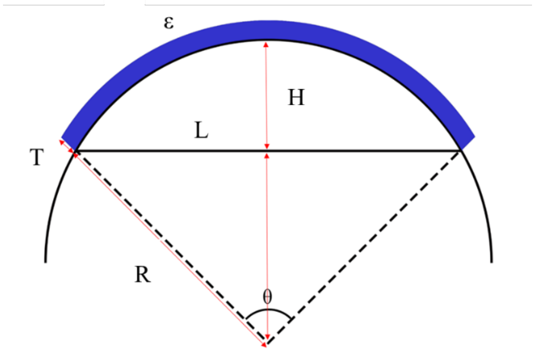

Supplementary Fig. 8. Calculation of the bending strain when bending the device to different strains, $\varepsilon = T/2R$, where T is the combined thickness of the sensing film and substrate, and R is the bending radius.

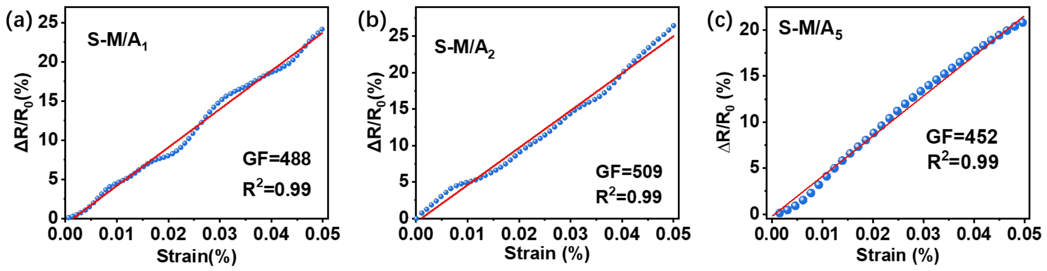

Supplementary Fig. 9. Relative resistance variation as a function of tiny strain (0–0.05%) for (a) S-M/A_1, (b) S-M/A_2, and (c) S-M/A_5 sensors.

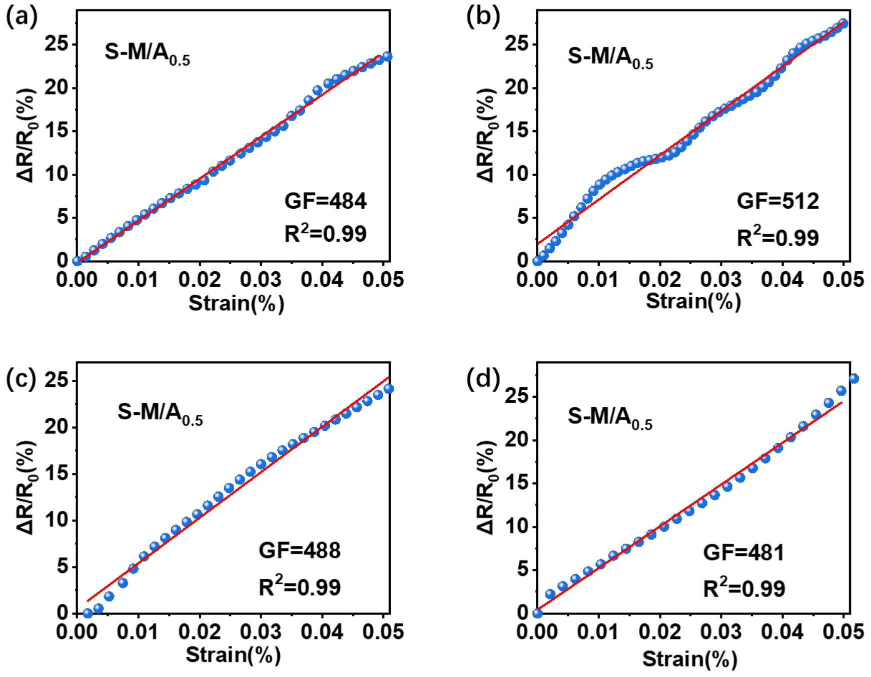

Supplementary Fig. 10. The other 4 repeated results of relative resistance variation as a function of tiny strain (0–0.05%) for the S-M/$A_{0.5}$ sensor.

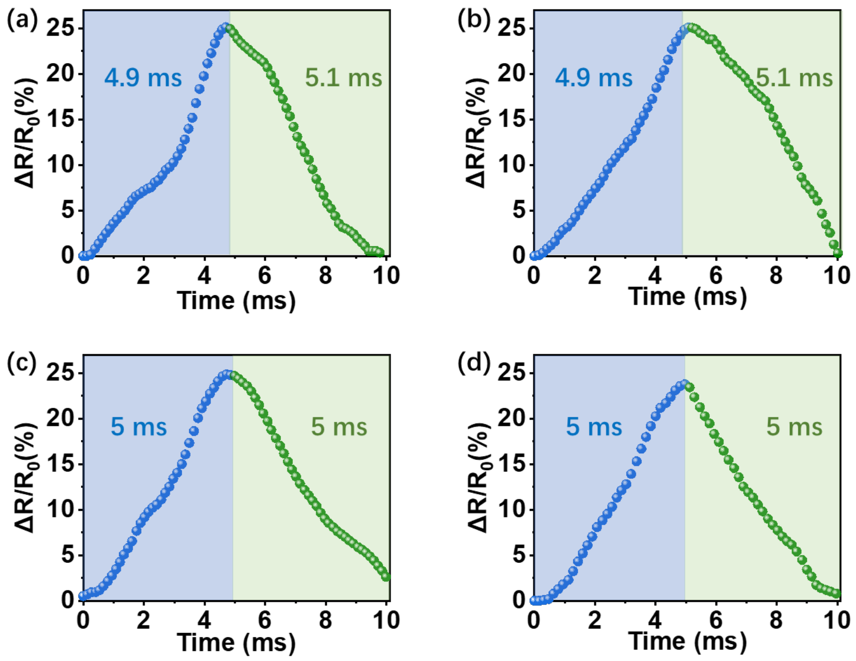

Supplementary Fig. 11. The other 4 repeated results of the transient sensing response and recovery time to an applied strain of 0.05% for the S-M/$A_{0.5}$ sensor.

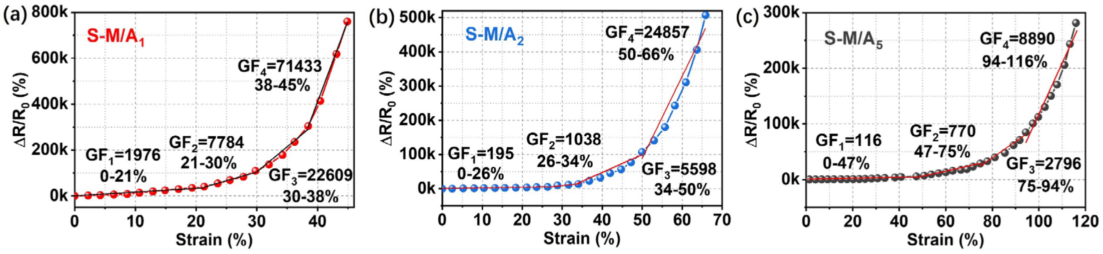

Supplementary Fig. 12. Relative resistance variation as a function of large strain for (a) S-M/A$_1$, (b) S-M/A$_2$, and (c) S-M/A$_5$ sensors.

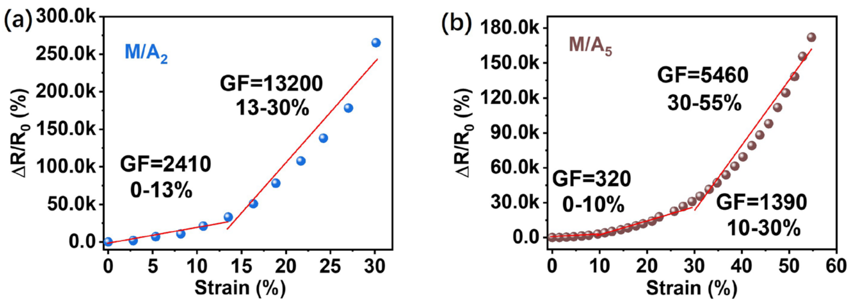

Supplementary Fig. 13. Relative resistance variation as a function of large strain for (a) M/A$_2$ and (b) M/A$_5$ sensors.

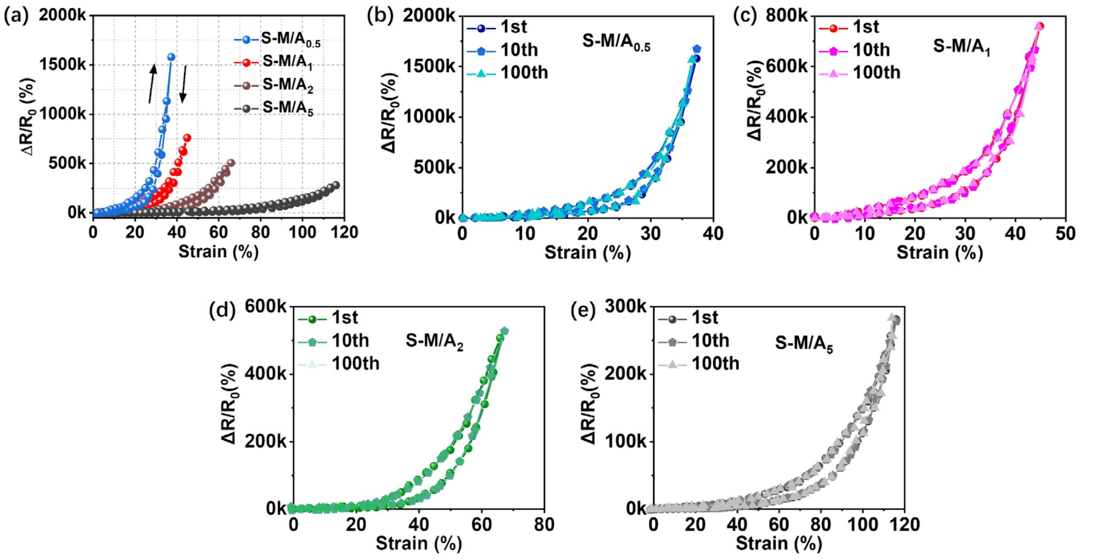

Supplementary Fig. 14. (a) Relative resistance variation as a function of large strain for S-M/A$_{0.5}$, S-M/A$_1$, S-M/A$_2$, and S-M/A$_5$ sensors under one stretch-release cycle. Detailed relative resistance changes versus strain curves at 1st, 10th, and 100th stretch-release cycle for (b) S-M/A$_{0.5}$, (c) S-M/A$_1$, (d) S-M/A$_2$, and (e) S-M/A$_5$ sensors.

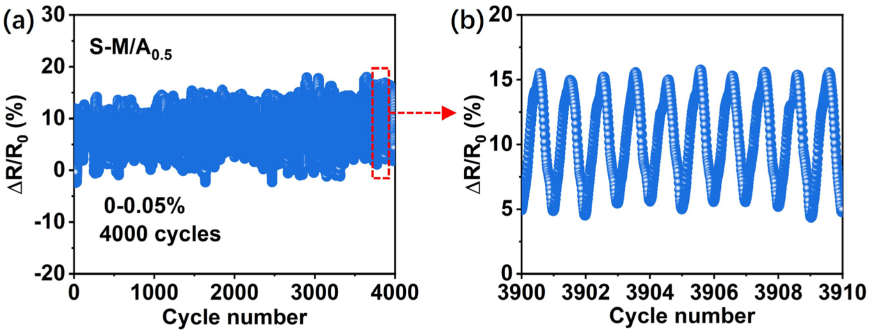

Supplementary Fig. 15. (a) Relative resistance changes of the S-M/$A_{0.5}$ sensor over 4000 stretch-release cycles in the strain range of 0–0.05%. (b) Detailed resistance changes recorded between 3900 and 3910 stretch-release cycles.

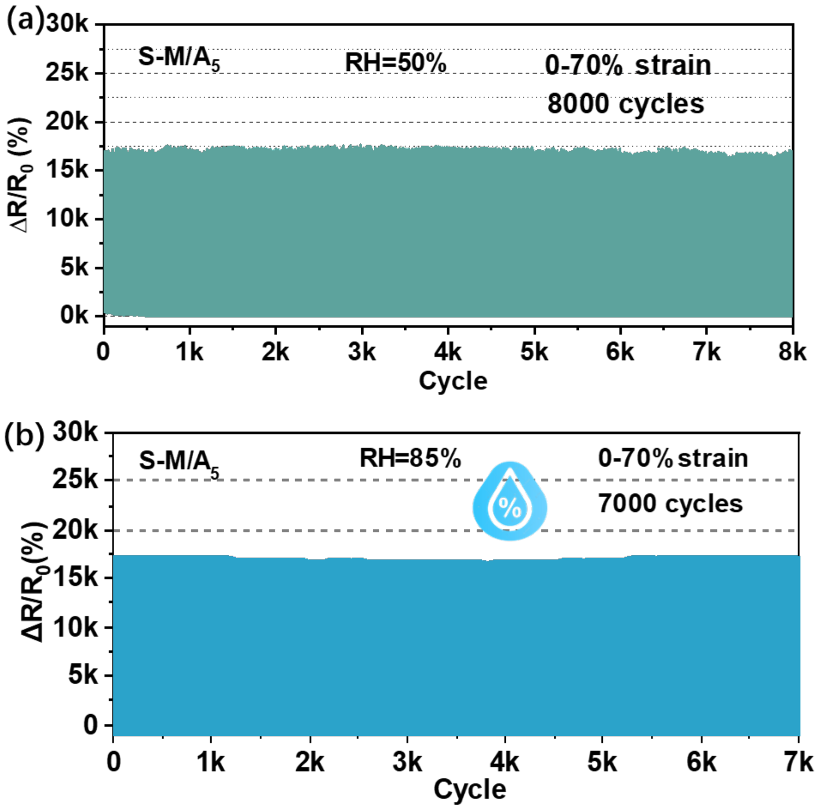

Supplementary Fig. 16. Relative resistance changes of S-M/A_5 sensor (a) over 8000 stretch-release cycles in the strain range of 0–70% under RH of 50% and (b) over 7000 stretch-release cycles in the strain range of 0–70% under RH of 85%.

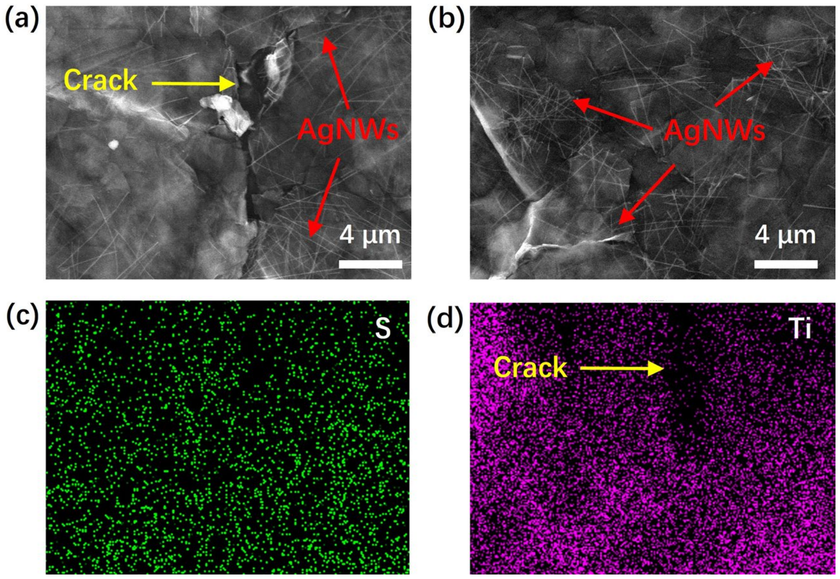

Supplementary Fig. 17. (a) and (b) Magnifed SEM images of S-M/A film under 60% strain. EDS element maps of (c) S and (d) Ti from (a). Uniform distribution of AgNWs can be clearly seen in the S-M/A film.

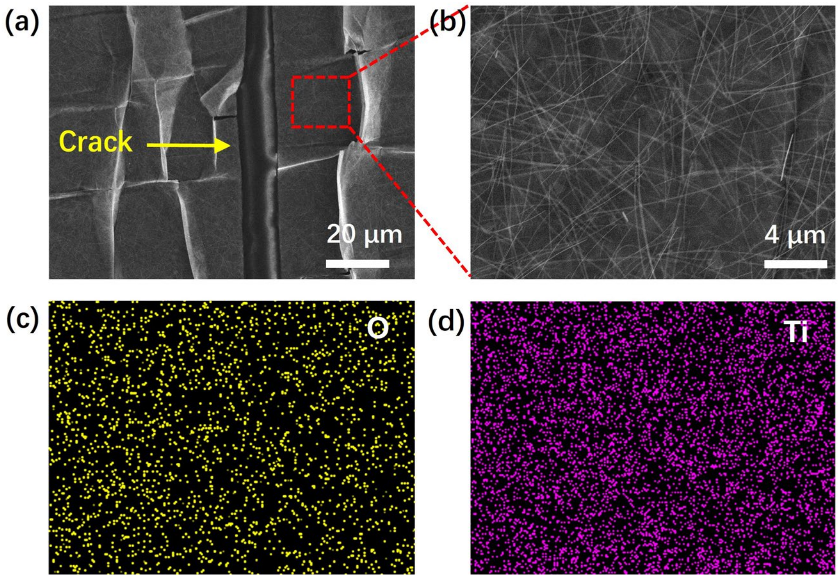

Supplementary Fig. 18. (a) and (b) SEM images of M/A film under 60% strain. EDS element maps of (c) O and (d) Ti from (b). Uniform distribution of AgNWs can be clearly seen in the M/A film.

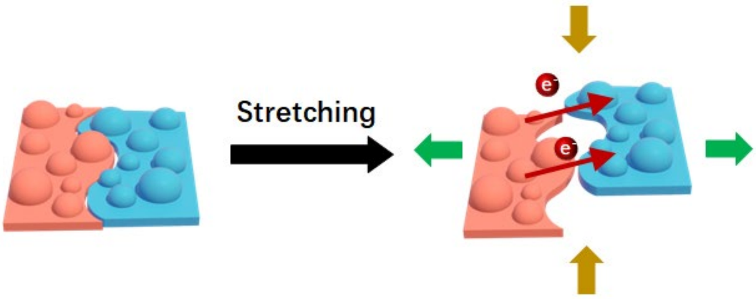

Supplementary Fig. 19. Schematic diagram of the proposed working principle of the tunneling effect for the crack-based S-M/A strain sensor.

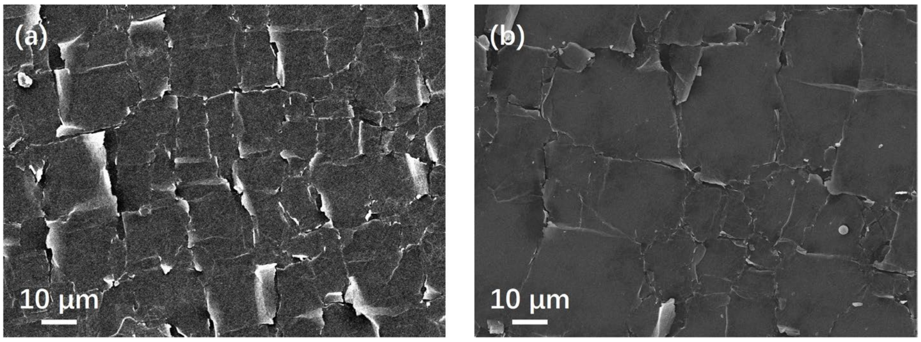

Supplementary Fig. 20. SEM images of S-M/A sensing film (a) under 100% strain and (b) after 5000 stretch-release cycles at 60% strain.

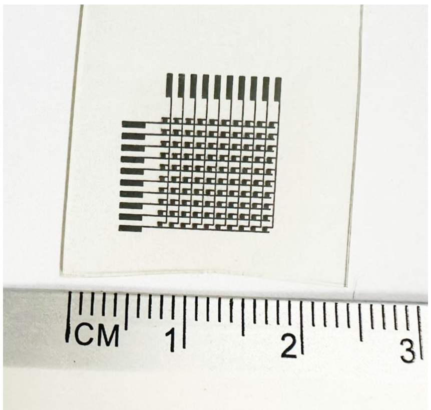

Supplementary Fig. 21. Photograph of a 100-channel S-M/$A_{0.5}$ strain sensor array with a device density of 100 sensors per square centimeter.

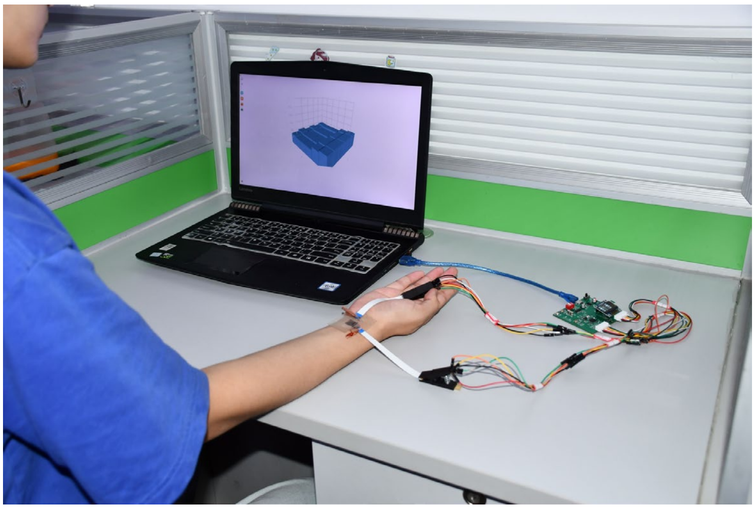

Supplementary Fig. 22. Photograph showing the monitoring of an artery pulse waveform of a volunteer using our multichannel pulse sensing system.

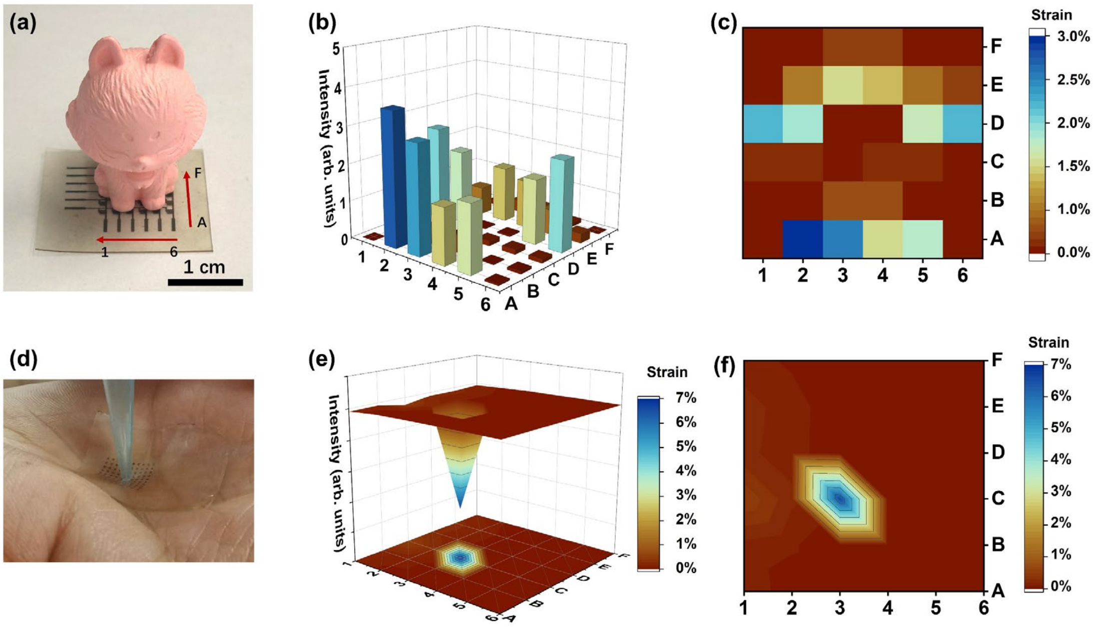

Supplementary Fig. 23. (a) Photograph of a 36-channel S-M/A_1 strain sensor array detecting a small object with complex shape, and (b) and (c) the corresponding intensity distribution of the normalized resistance change on the sensing array. (d) Photograph of a pipette tip poking on a 36-channel S-M/A_1 strain sensor array, and (e) and (f) the corresponding intensity distribution of the normalized resistance change on the sensing array.

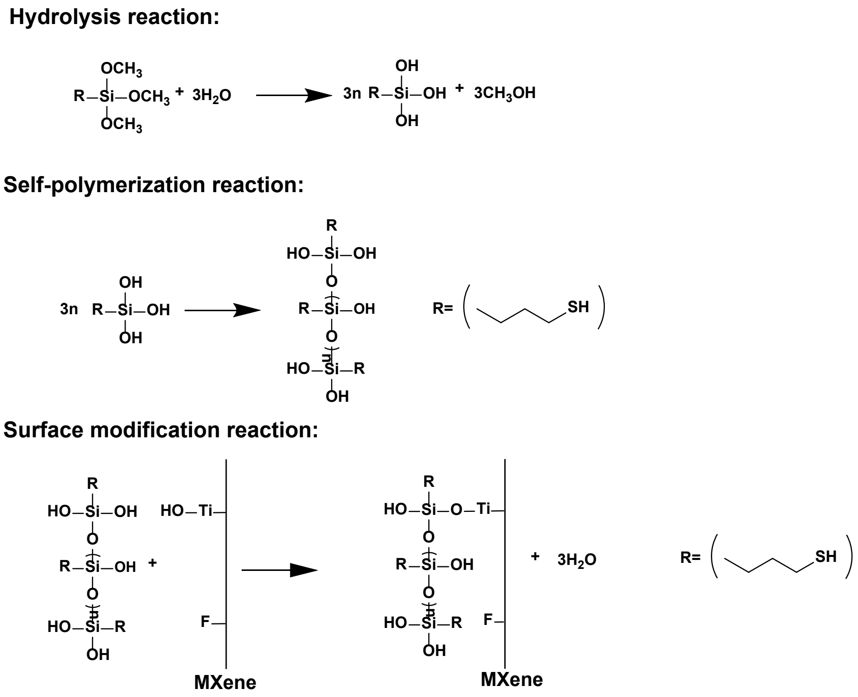

Supplementary Fig. S24. The hydrolysis, self-polymerization, surface modification reaction mechanism of MPTES.

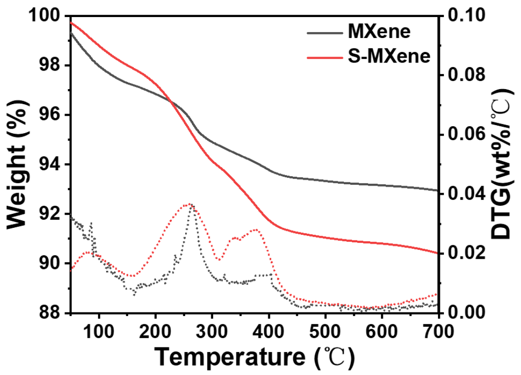

Supplementary Fig. S25. TGA and DTG results for S-MXene and MXene. The content of MPTES grafted onto the S-MXene was about 3.6 wt%.

Supplementary Table 1.

Size (mm^2)	Minimum detectable strain (%)	Maximum detectable strain (%)	Maximum Gauge factor	Response and Recovery time (ms)	Reference

0.25	0.001	37	152500	NA	This work
4	0.001	66	24900	NA	This work
25	0.001	116	8890	NA	This work
78.5	0.01	44	108241.7	NA	7
200	NA	100	12274	NA	8
75	0.000064	1	8699	0.107 @ 0.58 (0.1%)	9
56.25	NA	3	85000	NA	10
200	NA	145	42300	NA	11
50	NA	2	2000	NA	12
2000	0.1	170	1989	150 @ 150 (100%)	13
150	NA	100	363	NA	14
105	NA	2	16000	NA	15
30	NA	1	5000	NA	16
100	0.1	130	772.6	NA	17
120	0.025	74.1	1148.2	NA	18
60	NA	83	8700	NA	19
300	0.2	2	22.6	60 @ 60 (0.28%)	20
NA	0.006	0.8	450	NA	21
400	0.005	6.5	18000	258 @ 247 (0.65%)	22
224	0.5	0.9	1001	140 @ 228 (0.61%)	23
176.6	33	930	810	NA	24

Supplementary Movie 1: Real-time and dynamic display of the 3D pulse strength distribution measured by the 36-channel S-M/A_1 strain sensor array.

Supplementary References

1. Choi, W. Y., *et al.* Ultra-sensitive Pressure sensor based on guided straight mechanical cracks. *Sci. Rep.* **7**, 40116 (2017).

2. Cao, J, *et al.* A universal and facile approach to suppress dendrite formation for a Zn and Li metal anode. *J. Mater. Chem. A* **8**, 9331-9344 (2020).

3. Firestein, K. L., *et al.* Young's Modulus and Tensile Strength of Ti_3C_2 MXene Nanosheets As Revealed by In Situ TEM Probing, AFM Nanomechanical Mapping, and Theoretical Calculations. *Nano Lett.* **20**, 5900-5908 (2020).

4. Bernal, R. A., *et al.* Intrinsic Bauschinger Effect and Recoverable Plasticity in Pentatwinned Silver Nanowires Tested in Tension. *Nano Lett.* **15**, 139-146 (2015).

5. Echols, I. J., *et al.* Electronic and Optical Property Control of Polycation/MXene Layerby-Layer Assemblies with Chemically Diverse MXenes. *Langmuir* **37**, 11338-11350 (2021).

6. McCarthy, E. K., Bellew, A. T., Sader, J. E., Boland, J. J. Poisson's ratio of individual metal nanowires. *Nat. Commun.* **5**, 7 (2014).

7. Wang, L., *et al.* Crack sensing of cardiomyocyte contractility with high sensitivity and stability. *ACS Nano* **16**, 12645-12655 (2022).

8. Kim, K., Hong, S. K., Ha, S., Li, L., Lee, H. W., Kim, J. Enhancement of linearity range of stretchable ultrasensitive metal crack strain sensor via superaligned carbon nanotube-based strain engineering. *Mater. Horiz.* **7**, 2662-2672 (2020).

9. Dinh Le, T., *et al.* Ultrasensitive anti-interference voice recognition by bio-inspired skin-attachable self-cleaning acoustic sensors. *ACS Nano* **13**, 13293-13303 (2019).

10. Araromi, O. A., *et al.* Ultra-sensitive and resilient compliant strain gauges for soft machines. *Nature* **587**, 219-224 (2020).

11. Lee, J., Pyo, S., Kwon, D., Jo, E., Kim, W., Kim, J. Ultrasensitive strain sensor based on separation of overlapped carbon nanotubes. *Small* **15**, 1805120 (2019).

12. Kang, D., *et al.* Ultrasensitive mechanical crack-based sensor inspired by the spider sensory system. *Nature* **516**, 222-226 (2014).

13. Lin, J., *et al.* Anti-liquid-interfering and bacterially antiadhesive strategy for highly stretchable and ultrasensitive strain sensors based on Cassie-Baxter wetting state. *Adv. Funct. Mater.* **30**, 2000398 (2020).

14. Wang, H., *et al.* High-Performance Foam-Shaped Strain Sensor Based on Carbon Nanotubes and $Ti_3C_2T_x$ MXene for the Monitoring of Human Activities. *ACS Nano* **15**, 9690-9700 (2021).

15. Park, B., *et al.* Dramatically enhanced mechanosensitivity and signal-to-noise ratio of nanoscale crack-based sensors: effect of crack depth. *Adv. Mater.* **28**, 8130-8137 (2016).

16. Yang, T., *et al.* Structural engineering of gold thin films with channel cracks for ultrasensitive strain sensing. *Mater. Horiz.* **3**, 248-255 (2016).

17. Cai, Y., *et al.* Stretchable $Ti_3C_2T_x$ MXene/carbon nanotube composite based strain sensor with ultrahigh sensitivity and tunable sensing range. *ACS Nano* **12**, 56-62 (2018).

18. Yang, Y., *et al.* $Ti_3C_2T_x$ MXene-graphene composite films for wearable strain sensors featured with high sensitivity and large range of linear response. *Nano Energy* **66**, 104134 (2019).

19. Shi, X., *et al.* Bioinspired ultrasensitive and stretchable MXene-based strain sensor via nacre-mimetic microscale "brick-and-mortar" architecture. *ACS Nano* **13**, 649-659 (2018).

20. Cai, Y., *et al.* Graphdiyne-Based Nanofilms for Compliant On-Skin Sensing. *ACS Nano* **16**, 16677-16689 (2022).

21. Wang, Y., *et al.* Two-dimensional mechano-thermoelectric heterojunctions for self-powered strain sensors. *Nano Lett.* **21**, 6990-6997 (2021).

22. Liu, L., *et al.* Bioinspired, omnidirectional, and hypersensitive flexible strain sensors. *Adv. Mater.* **34**, 2200823 (2022).

23. Wang, D., *et al.* A selective-response bioinspired strain sensor using viscoelastic material as middle layer. *ACS Nano* **15**, 19629-19639 (2021).

24. Yang, H., *et al.* Wireless $Ti_3C_2T_x$ MXene strain sensor with ultrahigh sensitivity and designated working windows for soft exoskeletons. *ACS Nano* **14**, 11860-11875 (2020).
