# Supplementary material for: Ag–thiolate interactions to enable an ultrasensitive and stretchable MXene strain sensor with high temporospatial resolution
Source: Nat Commun. 2024 Jun 25;15:5354. doi: 10.1038/s41467-024-49787-9 (PMC11200319; doi:10.1038/s41467-024-49787-9)
Supplement: Supplementary file 4 — Description of Additional Supplementary Files [file 41467_2024_49787_MOESM4_ESM.pdf]

Description of Additional Supplementary Files

File Name: Supplementary Movie 1

Description: Real-time and dynamic display of the 3D pulse strength distribution measured by the 36-channel S-M/A1 strain sensor array.
